# Metagenomic quorum quenching enzymes affect biofilm formation of *Candida albicans* and *Staphylococcus epidermidis*

**DOI:** 10.1371/journal.pone.0211366

**Published:** 2019-01-28

**Authors:** Nancy Weiland-Bräuer, Irene Malek, Ruth A. Schmitz

**Affiliations:** Kiel University, Institute for General Microbiology, Kiel, Germany; Louisiana State University, UNITED STATES

## Abstract

Biofilm formation in the clinical environment is of increasing concern since a significant part of human infections is associated, and caused by biofilm establishment of (opportunistic) pathogens, for instance *Candida albicans* and *Staphylococcus epidermidis*. The rapidly increasing number of antibiotic-resistant biofilms urgently requires the development of novel and effective strategies to prevent biofilm formation ideally targeting a wide range of infectious microorganisms. Both, synthesis of extracellular polymeric substances and quorum sensing are crucial for biofilm formation, and thus potential attractive targets to combat undesirable biofilms.We evaluated the ability of numerous recently identified metagenome-derived bacterial quorum quenching (QQ) proteins to inhibit biofilm formation of *C*. *albicans* and *S*. *epidermidis*. Here, proteins QQ-5 and QQ-7 interfered with the morphogenesis of *C*. *albicans* by inhibiting the yeast-to-hyphae transition, ultimately leading to impaired biofilm formation. Moreover, QQ5 and QQ-7 inhibited biofilm formation of *S*. *epidermidis;* in case of QQ7 most likely due to induced expression of the *icaR* gene encoding the repressor for polysaccharide intercellular adhesin (PIA) synthesis, the main determinant for staphylococcal biofilm formation. Our results indicate that QQ-5 and QQ-7 are attractive potential anti-biofilm agents in the prevention and treatment of *C*. *albicans* and *S*. *epidermidis* mono-species biofilms, and potentially promising anti-biofilm drugs in also combating multi-species infections.

## Introduction

Bacterial biofilms are a major threat to human health as they are inherently resistant to clearance by both the host immune system and antibiotics [1]. Moreover, biofilms are often multispecies consortia formed from members of the endogenous microbiota as well as nosocomial pathogens. These biofilms can be difficult to detect as well as to treat, especially when prokaryotes and eukaryotes, e.g. bacteria and fungi co-occur, mostly requiring complex multi-drug treatment strategies [2]. *Candida albicans* is the most prevalent human fungal pathogen asymptomically inhabiting diverse host niches. However, it is also able to cause disease in both, immune-competent and immune-compromised individuals. Thus, *Candida* biofilms on indwelling medical devices and mucosal tissues are one of the most common causes of systemic lethal infections [3]. The coagulase-negative staphylococci, in particular *Staphylococcus epidermidis*, have likewise emerged as major nosocomial bacterial pathogens associated with infections of implanted medical devices [4, 5], often requiring a removal of an indwelling medical device [6]. As fungi and bacteria co-inhabit a wide range of environments, it is not surprising that *Candida* and various bacteria also form multispecies biofilms [7]. Interactions between fungi and bacteria ranging from antagonism to commensalism can have dramatic effects on the survival, colonization and pathogenesis of both organisms. Thus, those mixed fungal–bacterial biofilms can have properties that are distinct from their single-species counterparts. For instance, *Candida spp*. have been demonstrated to form biofilms with *Staphylococcus epidermidis in vivo* [8, 9]. Catheter disk models with such mixed biofilms demonstrated an altered, impaired sensitivity of each species to antimicrobial agents as a result of their mutual interaction [10].

Since antibiotics are frequently losing their effectiveness due to evolving antibiotic—or even multi-drug resistance, new antimicrobial strategies have to be considered and developed to prevent bacterial as well as fungal biofilm formation. Finding treatments altering the phenotype of the pathogen without selecting for viability of the species, which might lead to resistance, is a promising strategy in combating harmful biofilms [11]. One of the cellular processes crucial for biofilm formation, pathogenicity and virulence is cell-cell communication (quorum sensing, QS). Consequently, QS might be an attractive and most likely effective option for alternative novel drug design in medical as well as industrial applications [12]. The mechanisms causing the inactivation of QS systems are generally known as “quorum sensing interference” (QSI) or “quorum quenching” (QQ) [13–15]. The QS interference can be achieved by affecting QS molecule synthesis, inhibition of QS molecule/receptor interaction; and modification or degradation of signaling molecules as well as by the release of antagonistic small molecules. Syntheses of QS interfering compounds have been demonstrated for bacteria as well as for eukaryotes. Consequently, in recent years numerous QS interfering enzymes and small molecules have been screened and identified in chemical substance libraries, extracts of pure bacterial cultures isolated from eukaryotes as well as from metagenomic clone libraries [14–19]. Thus, naturally occurring QQ biomolecules are already used as novel therapeutic agents combating antibiotic- resistant microorganisms (reviewed in [20]).

The goal of this study was to evaluate the effects of numerous previously identified QQ active proteins [18] on biofilm formation of *C*. *albicans* as well as *S*. *epidermidis*, two of the most common causes of nosocomial infections. Two promising metagenome-derived QQ proteins, QQ-5 and QQ-7, are presented as novel biotechnologically relevant anti-pathogenic compounds, which are able to inhibit both, bacterial as well as fungal biofilm formation most likely by interfering different QS systems.

## Results

We recently identified nine QQ enzymes by functionally screening various metagenomic large insert libraries [18, 19]. The purified QQ proteins were able to interfere with acyl homoserine lactone (AHL)- and/or autoinducer-2 (AI-2) based cell-cell communication, and have been demonstrated to ultimately prevent biofilm formation of Gram-negative and Gram-positive biofilm forming model organisms (*Escherichia coli*, *Pseudomonas aeruginosa*, *Klebsiella oxytoca*, *Bacillus subtilis*, *S*. *aureus*) *in vitro* and *in vivo* [18]. All identified QQ proteins were initially screened for their ability to interfere with *in vitro* biofilm formation of *C*. *albicans* in 12 well multiwell plates (MWPs) and of *S*. *epidermidis* in 96 well microtiter plates (MTPs). Proteins QQ-5 and QQ-7 were identified as potentially potent proteins to interfere *C*. *albicans* and *S*. *epidermidis* biofilm formation, and were further characterized.

### Delayed and reduced biofilm formation of *C*. *albicans* in presence of QQ-5 and QQ-7

**Fig 1:**
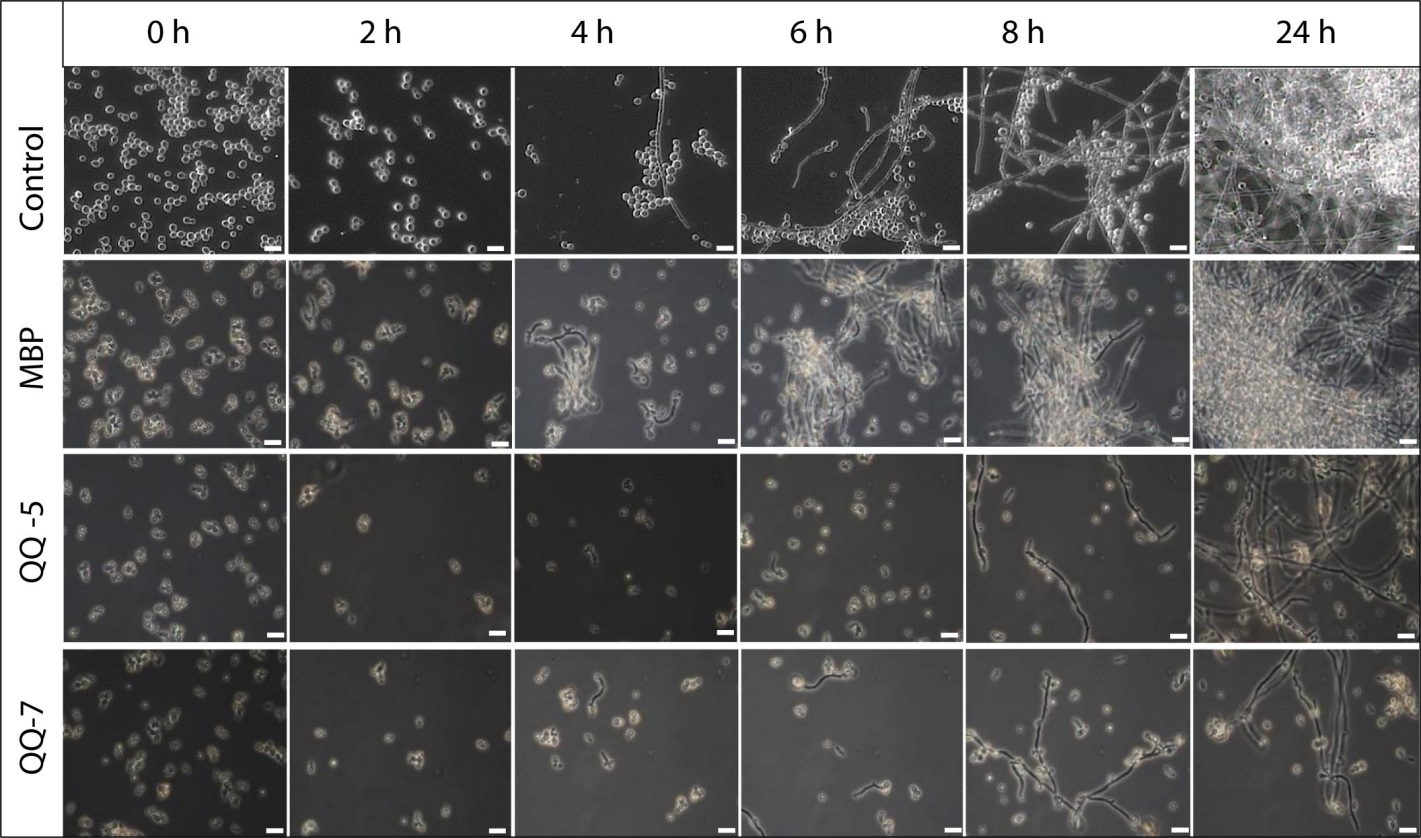
Effects of immobilized QQ proteins on the biofilm formation of *C*. *albicans*. Phase-contrast images were taken with a Zeiss Axioscope microscope over a time period of 2 to 24 h. QQ proteins MBP-QQ-5 and MBP-QQ-7 as well as MBP were immobilized in 12 well culture dishes in comparision to the control. *C*. *albicans* cells (10^9^ yeast cells/mL) were incubated in YPD at 30°C to analyze yeast to hyphae formation. Scale bars represent 10 μm.

Biofilm formation of *C*. *albicans* was monitored in 12 well MWPs over a 24 h period by phase-contrast microscopy, and effects of immobilized QQ proteins on biofilm formation were elucidated. In controls without supplement or immobilized control protein Maltose binding protein (MBP), *C*. *albicans* cells rapidly attached to the surface and formed germ tubes after 2 h of incubation (Fig 1, two upper panels). First formation of long, branching filaments, so called hyphae, was detected after 4 h. Subsequent proliferation of yeast cells and formation of hyphae resulted in a dense network of yeast cells, hyphae and extracellular polymeric matrix forming a mature *Candida* biofilm within 24 h (Fig 1, two upper panels). However, in the presence of immobilized purified MBP-QQ fusion proteins, proliferation of yeast cells was inhibited and germ tube formation was delayed. First hyphae were detected after 8 h, and consequently the dense network of yeast cells and hyphae was not formed within 24 h. Thus, the transition from yeast-to-hyphae was extensively affected by the QQ proteins resulting in reduced biofilm formation after 24 h (Fig 1, two lower panels).

**Fig 2:**
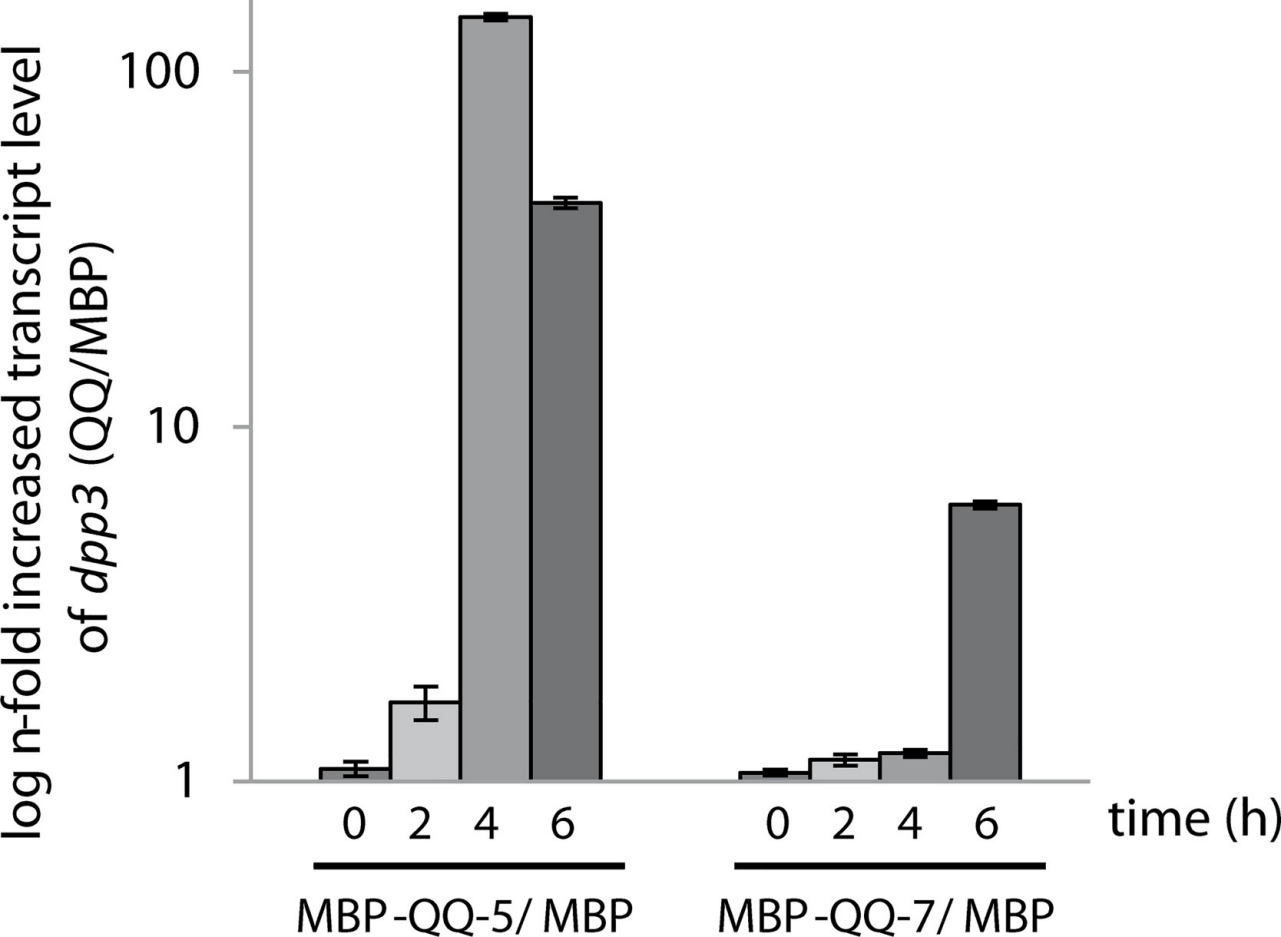
Relative transcript levels of *dpp3* in *C*. *albicans* after incubation with QQ proteins. Relative transcript levels of *dpp3* were determined by quantitative RT-PCR analysis in *C*. *albicans* with at least three biological replicates each with three technical replicates. QQ proteins MBP-QQ-5 and MBP-QQ-7 as well as MBP were immobilized on the surface of culture dishes. *C*. *albicans* cells (10^9^ yeast cells/mL) were incubated for 2 h to 6 h at 30°C in YPD. Fold changes in transcript abundances were determined by comparison with the threshold cycle (Ct) of transcripts (calculated 2^-ΔΔCt^ values) of the control gene *actin*.

Furthermore, effects of the QQ proteins on the transcription of the key gene *dpp3*, involved in farnesol synthesis, were analyzed by qRT-PCR demonstrating that transcript levels of *dpp3* were significantly upregulated in the presence of immobilized MBP-QQ-5 and MBP-QQ-7 (Fig 2). In more detail, after 2 h, increased *ddp3* transcript levels were detected in the presence of MBP-QQ-5, with highest levels after 4 h of incubation (150-fold increase) most likely resulting in increased farnesol synthesis. Since high concentrations of farnesol prevent the switch to generate hyphal filaments this consequently results in delayed biofilm formation (Fig 2, left panel). In the presence of immobilized MBP-QQ-7, *dpp3* transcription was only slightly upregulated after 6 h of incubation (6 fold increase) (Fig 2, right panel) supporting the microscopy analysis showing a minor effect on hyphae formation at this time point (Fig 1). In conclusion, these findings argue for the inhibition of the yeast-to-hyphae transition by QQ-5 and QQ-7 presumably involving stimulated farnesol production through upregulation of *dpp3*, and thus leading to delayed biofilm formation.

### Effects of QQ-5 and QQ-7 on *S*. *epidermidis* biofilm formation in batch cultures

**Fig 3:**
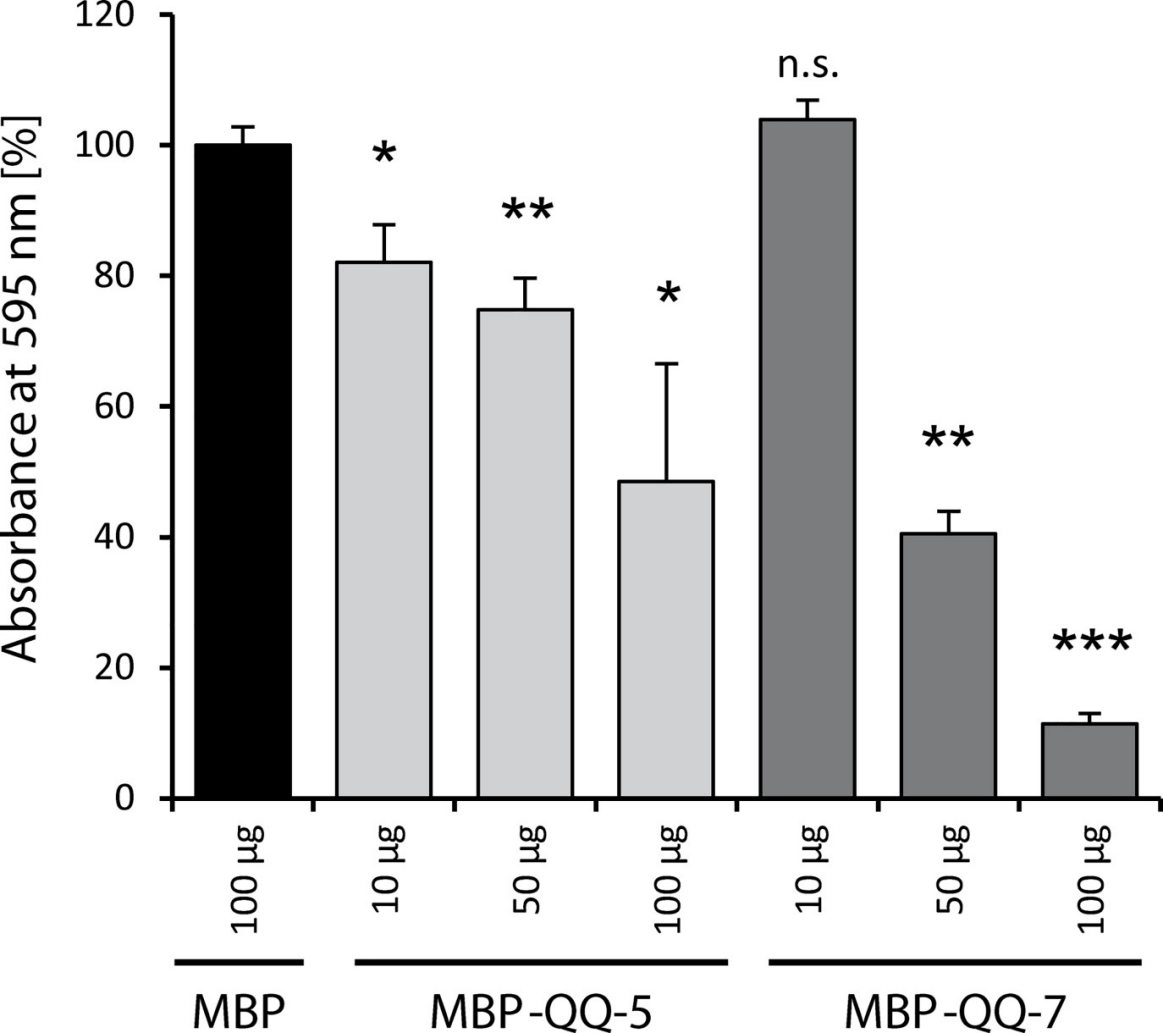
*S*. *epidermidis* biofilm formation under static conditions in presence of MBP-QQ-5, MBP-QQ-7 and MBP. *S*. *epidermidis* was grown in 96 well plates in 200 μL culture medium in presence of purified MBP-QQ proteins MBP-QQ-5 (light grey bars) and MBP-QQ-7 (dark grey bars) at final concentrations of 10 μg, 50 μg and 100 μg. Purified MBP (black bar) was added as control to a final concentration of 100 μg and the determined absorbance set to 100%. Biofilm formation was quantified via crystal violet assay. Means of standard deviations of at least 4 independent biological replicates are indicated. Statistics (unpaired *t* test) were performed with GraphPad Prism 6 software with differences *p < 0.05, **p < 0.01 and ***p < 0.001 considered significant; n.s., not significant.

Biofilm formation of *S*.*epidermidis* was analyzed in 200 μL batch cultures in 96 well MTPs after 24 h of incubation under static conditions in the presence of MBP (control) and increasing concentrations of purified MBP-QQ-5 as well as MBP-QQ-7 using the crystal violet assay [21, 22]. Biofilm formation significantly decreased in the presence of increasing MBP-QQ-5 concentrations (Fig 3). Compared to the MBP control (100% equal); biofilm formation was reduced to 82 ± 6% in the presence of 10 μg MBP-QQ-5. The presence of 50 μg and 100 μg MBP-QQ-5 resulted in significant biofilm reduction to 75 ± 5% and 48 ± 18% , respectively. 10 μg MBP-QQ-7 had no effect on biofilm maturation; however a significant reduction to 41 ± 3% and 11 ± 2% was obtained in the presence of 50 μg and 100 μg MBP-QQ-7, respectively (Fig 3).

### Effects of immobilized QQ proteins on *S*. *epidermidis* biofilm formation in continuous flow chambers

**Fig 4:**
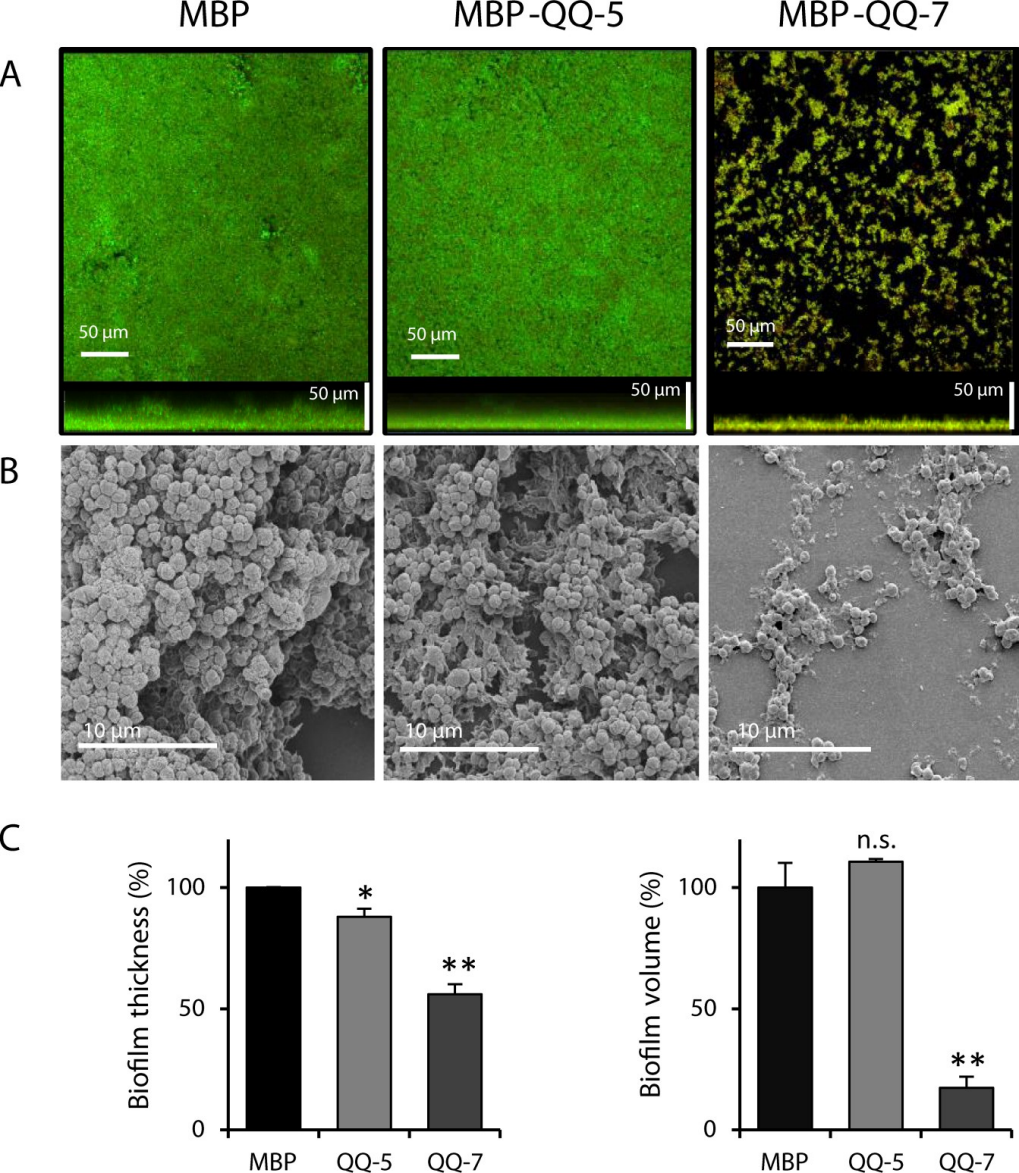
Decreased *S*. *epidermidis* biofilm formation on immobilized proteins MBP-QQ-5 and MBP-QQ-7. Coverslips coated with proteins MBP-QQ-5, MBP-QQ-7and MBP as control were used in flow chambers (total volume of 1.3 mL) for *S*. *epidermidis* biofilm formation (3 x 10^8^ cells/ mL). After 1 h adhesion, flow chambers were incubated for 20 h (long-term experiment) with a flow rate of 20 mL/h using 3% TSB medium. Biofilms were stained with Syto9 and propidium iodide. Effects on biofilm formation were visualized using (***A***) CLSM and (***B*)** SEM. (***C***) Biofilm characteristics thickness and volume were quantified using IMARIS software. Means of 2 independent biological replicates, each with 3 technical replicates are indicated. Unpaired *t* test was performed with GraphPad Prism 6 software with differences *p < 0.05, **p < 0.01 and ***p < 0.001 considered significant.

Purified MBP-QQ-5, MBP-QQ-7 and MBP were immobilized on coverslips using the Layer-by-Layer method [23] prior to *S*. *epidermidis* biofilm formation in continuous flow chambers. Confocal Laser Scanning Microscopy (CLSM) was used to determine biofilm thickness and volume using the IMARIS software after 20 h of incubation. Thickness of biofilms, formed in the presence of immobilized MBP-QQ-5, was significantly reduced to 88 ± 3%; whereas the volume was not affected at all (111 ± 1%) (Fig 4A and 4C). However, immobilized MBP-QQ-7 revealed a substantial effect on biofilm formation of *S*. *epidermidis*. Biofilm thickness significantly decreased to 56 ± 4% and biofilm volume to 17 ± 5%. These drastic effects on biofilm maturation were confirmed by means of Scanning Electron Microscopy (SEM, Fig 4B). We further tested the effect of MBP alone and heat-treated (30 min 65°C), inactive MBP-QQ proteins immobilized to the coverslips on biofilm formation (20 h incubation) of *S*. *epidermidis*. Here, no effects on biofilm formation were observed (Table 1), demonstrating that most likely the enzyme activity and not anti-adhesive effects of the QQ proteins is responsible for inhibition of biofilm formation.

**Table 1:** Parameters of *S*. *epidermidis* biofilms in presence of heat-treated MBP-QQ proteins. Coverslips coated with heat-treated (30 min at 65°C) and non-treated proteins MBP-QQ-5, MBP-QQ-7and MBP as control were used in flow chambers for *S*. *epidermidis* biofilm formation (3 x 10^8^ cells/ mL). After 1 h adhesion, flow chambers were incubated for 20 h with a flow rate of 20 mL/h using 3% TSB medium. Biofilm characteristics thickness and volume were quantified using IMARIS software. Means and corresponding standard deviations of 2 independent biological replicates, each with 3 technical replicates are indicated.

	heat-treated			non heat-treated		
	MBP	MBP-QQ5	MBP-QQ7	MBP	MBP-QQ5	MBP-QQ7
**Thickness [μm]**	**42 ± 3**	**44 ± 4**	**39 ± 5**	**44 ± 4**	**42 ± 3**	**41 ± 4**
**Volume [**μ**m**^**3**^**/** μ**m**^**2**^**]**	**21 ± 3**	**23 ± 4**	**23 ± 2**	**22 ± 4**	**22 ± 3**	**21 ± 4**

**Fig 5:**
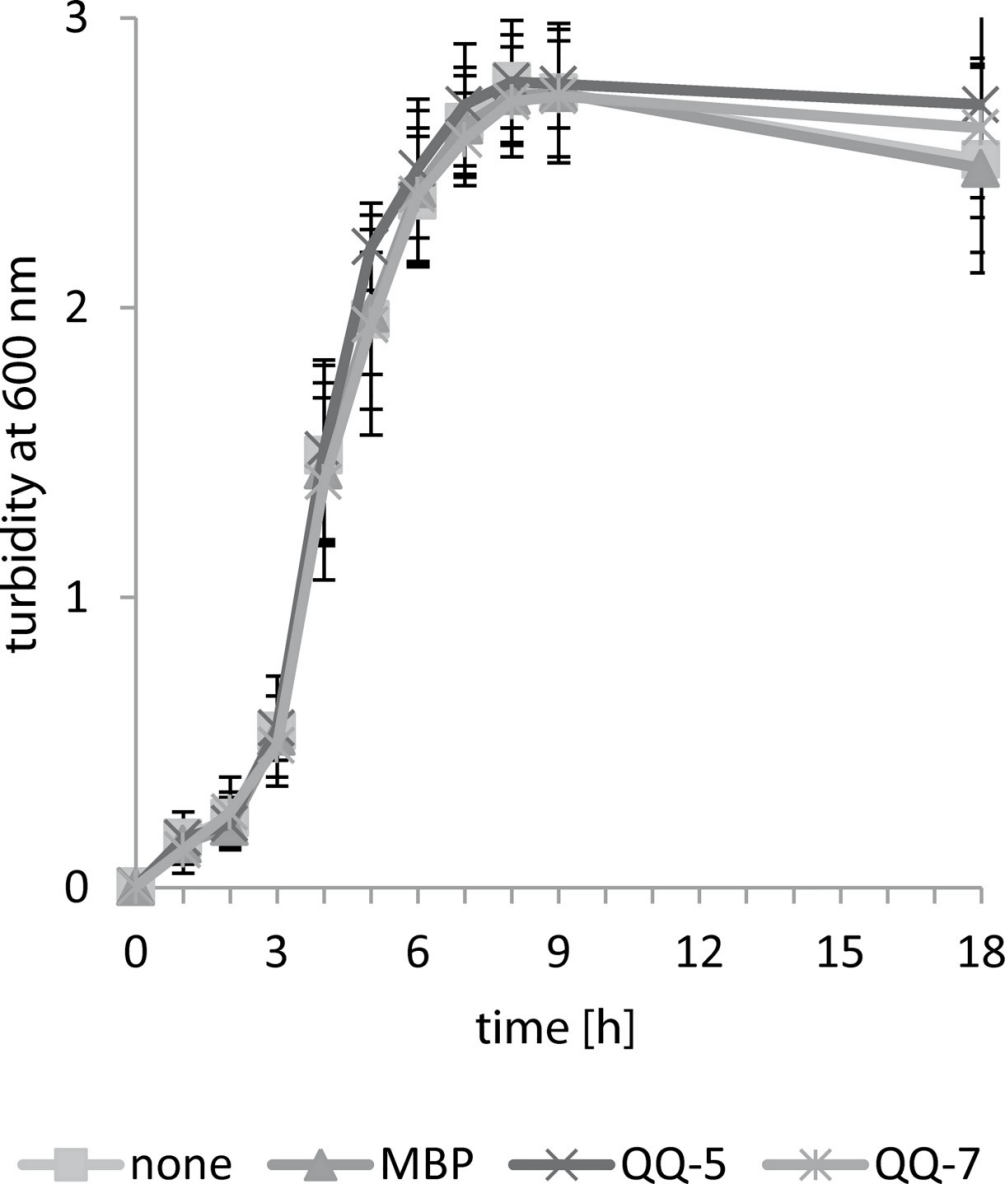
Excluding potential effects of QQ proteins on growth of *S*. *epidermidis* RP6A. *S*. *epidermidis* RP6A was grown in Tryptic Soy Broth medium at 37°C and 120 rpm in 20 mL. Cultures were grown complemented with 100 μg MBP, QQ-5 or QQ-7 as well as without any supplement. The results represent a mean of three independent experiments. Error bars indicate standard deviations.

Moreover, the ratio between living and dead cells within the biofilms was determined using CLSM and IMARIS software (Fig 4A) demonstrating that the number of living cells within biofilms formed on coverslips with immobilized MBP-QQ-5 was not affected (live/dead ratio of 1.68 compared to 1.47 when incubated with MBP). However, the live/dead ratio of biofilms established on immobilized MBP-QQ-7 was significantly lower (0.87) indicating a higher number of dead cells (Table 2), whereas growth in batch cultures was not affected by the presence of either QQ protein (Fig 5).

**Table 2:** Live/Dead ratios within *S*. *epidermidis* biofilms. Means of 2 independent biological replicates, each with 3 technical replicates are indicated. Unpaired t test was performed with GraphPad Prism 6 software with differences p < 0.05 considered as significant.

Incubation	Coating	Live/Dead ratio mean	Standard deviation	*P-*value (MBP/QQ)
Long-term	MBP	1.47	0.0082	
	QQ-5	1.68	0.0212	0.0058
	QQ-7	0.87	0.1719	0.0388
Short-term	MBP	1.80	0.0509	
	QQ-7	1.29	0.0771	0.0160

**Fig 6:**
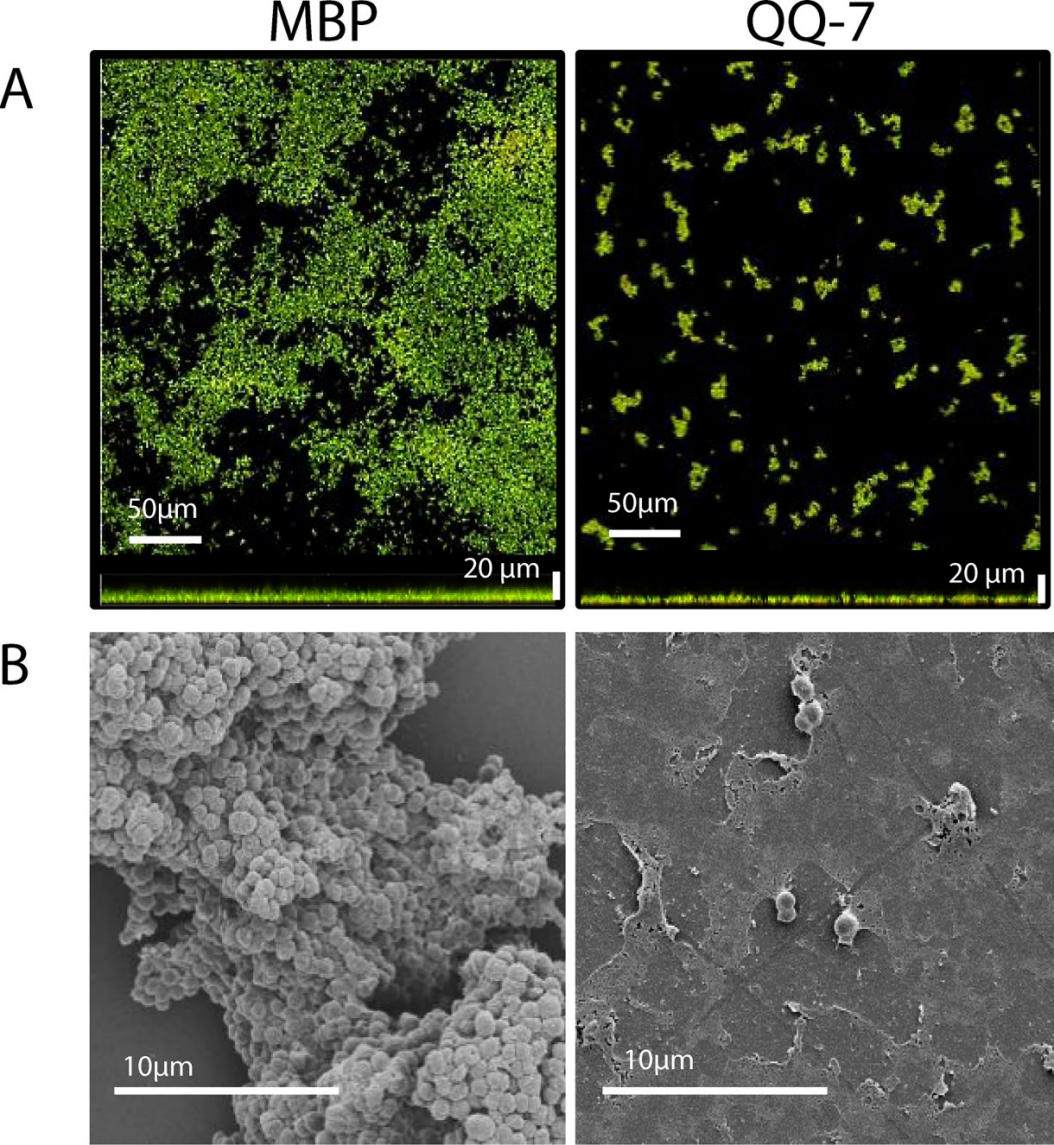
Impact of immobilized protein MBP-QQ-7 on *S*. *epidermidis* initial attachment. Proteins MBP-QQ-7 and MBP as control were immobilized on coverslips, which were inserted into flow chambers with a total volume of 1.3 mL. After 1 h adhesion, flow chambers were incubated for 3 h (short-term experiment) at a flow rate of 20 mL/h with 3% TSB medium. Adhered cells were stained with Syto9 and propidium iodide and visualized using (***A***) CLSM and (***B*)** SEM.

Additionally, a short-term flow chamber experiment (3 h incubation) was performed to determine a potential effect of immobilized MBP-QQ-7 on the early phase of biofilm formation (primary attachment and cell-cell adhesion). CLSM and SEM micrographs (Fig 6) clearly showed that attachment of cells was almost completely prevented in presence of active MBP-QQ-7. Analysis of the biofilm volume confirmed this result based on a significant decrease of the volume to 12% ± 3.5% and supporting our assumption that QQ-7 most likely interferes with biofilm formation at an early stage.

### Expression of *rnaIII*, *lrgA* and *icaR* in presence of QQ-7

**Fig 7:**
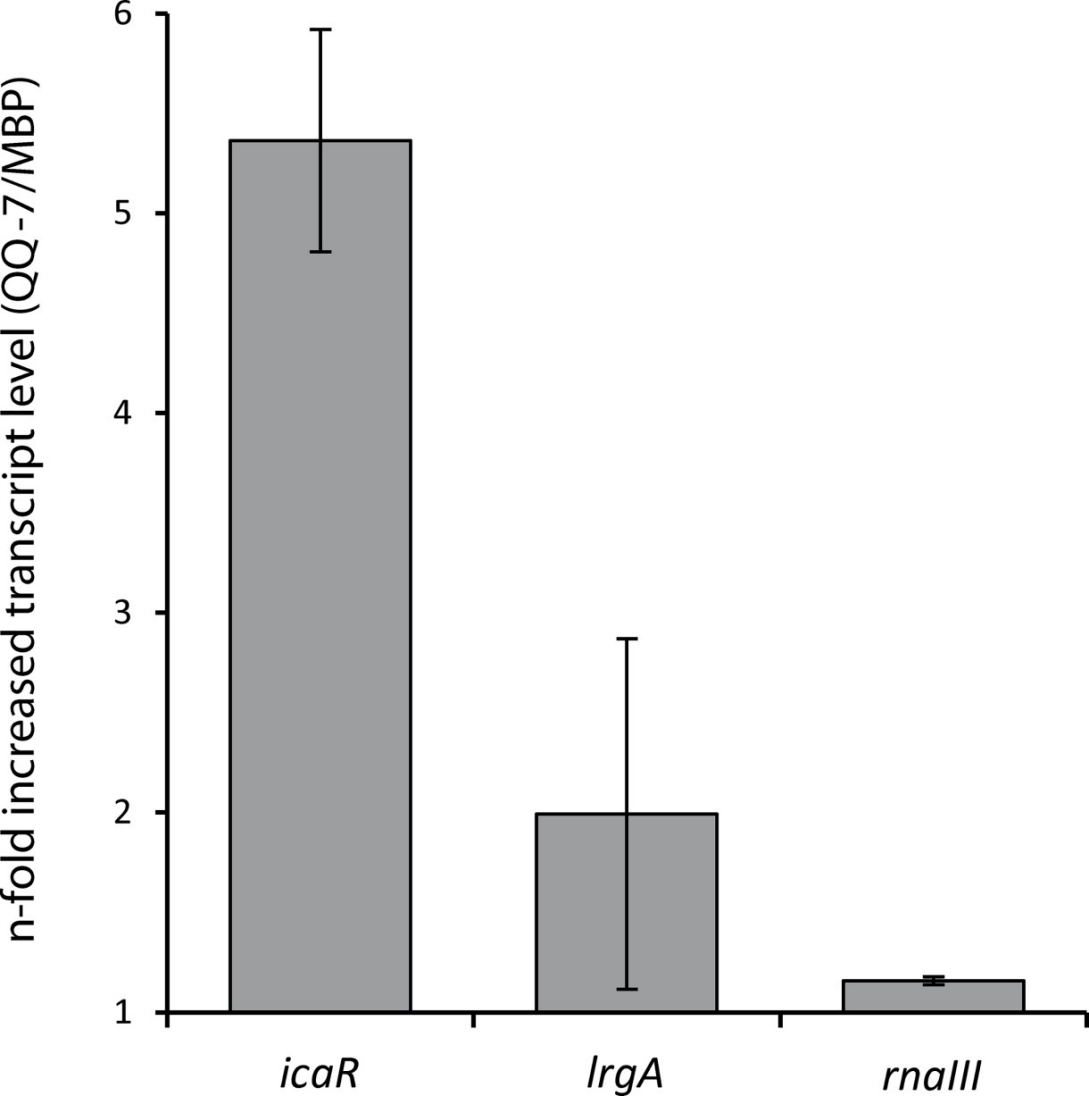
Transcript levels of *icaR*, *lrgA and rnaIII* in *S*. *epidermidis* in the presence of MBP-QQ-7. *S*. *epidermidis* (3 x 10^8^ cells/mL in 3 mL TSB) were incubated in the presence of 500 μg/mL MBP-QQ-7 without shaking. After 3 h incubation, RNA was isolated and the relative transcript levels of *icaR*, *lrgA* and *rnaIII* were determined by quantitative RT-PCR analysis. Three biological replicates each with three technical replicates were performed to determine the standard deviation of the calculated 2^-ΔΔCt^ values. Fold changes in transcript abundances were determined by comparison with the threshold cycle (Ct) of transcripts of the control gene *gyrB*.

Three factors involved in biofilm formation of *S*. *epidermidis* have been further elucidated concerning potential effects in response to QQ activity. Transcription of *rnaIII* (intracellular effector of the *agr* quorum sensing system) [24], the murein hydrolase regulator LrgA (mediator of murein hydrolase activity in *S*. *epidermidis*) [25] and the transcriptional regulator IcaR of the *icaADBC* operon [26] was analyzed by quantitative (q)RT-PCR. In the presence of MBP-QQ-7, *rnaIII* transcripts were not affected (1.16 ± 0.02 fold changed transcript level), *lrgA* transcript levels were slightly upregulated (1.99 ± 0.9 fold increased transcript level); whereas *icaR* transcript levels considerably increased (5.37 ± 0.6 fold) (Fig 7). Those results indicate interference with PIA synthesis by QQ-7 through repression of the *icaADBC* operon due to the upregulation of the IcaR repressor, consequently preventing *S*. *epidermidis* biofilm formation.

## Discussion

In the present study, the recently identified QQ active proteins QQ-5 and QQ-7 [18] were selected as promising candidates potentially affecting mono-species biofilm formation of *C*. *albicans* as well as *S*. *epidermidis*, ultimately demonstrating the transkingdom potential of QQ. Both enzymes are naturally occurring proteins previously identified in a metagenomic large insert library of a Salt Marsh (Hamburger Hallig/Germany) [18]. Both proteins originated from the marine Gram-negative bacterium *Nereida ignava* (α-Proteobacteria) and were able to simultaneously interfere with AHL- and AI-2 based QS as demonstrated in the reporter assay, most likely due to an oxidoreductase enzyme activity [18, 27]. Moreover, heterologous expression of both QQ-ORFs in *K*. *oxytoca* significantly affected AI-2 based biofilm formation of this medical relevant bacterium [18].

In the meantime, several studies disclosed that biofilm formation of both *C*. *albicans* as well as *S*. *epidermidis* is regulated through QS [28–32]. In *C*. *albicans*, biofilm formation is controlled by the QS molecule farnesol. Dpp3 phosphatase has been demonstrated to be involved in farnesol synthesis by converting farnesyl pyrophosphate to farnesol. High concentrations of farnesol prevent the switch from yeast cells to hyphal filaments, and consequently inhibit biofilm formation [32–35]. Our results from the present study indicate that QQ-5 and QQ-7 might also be able to affect farnesol synthesis in *C*. *albicans*. *Dpp3* expression was promoted by the QQ proteins (see Fig 2) concluding that present QQ proteins caused increased farnesol synthesis; ultimately preventing/delaying yeast-to-hyphae switch, and thus biofilm formation (see Fig 1). Biofilm formation of Staphylococci has been described as a multi-step process including primary attachment, cell-cell adhesion and accumulation [36], which is regulated by the QS systems *agr* and *luxS* [37]. Accessory gene regulator (*agr*)-mediated QS is mainly controlled by RNAIII, a regulatory RNA, which subsequently upregulates secreted virulence factors and downregulates cell surface proteins, thereby governing invasiveness of Staphylococci and cell dispersal from biofilms. LuxS was found to impact the expression of a series of biofilm-promoting genes, including biofilm exopolysaccharide biosynthesis [38]. Exopolysaccharides, such as the polysaccharide intercellular adhesin (PIA) are the main determinants for successful staphylococcal biofilm formation. PIA is encoded by the *ica* operon and is mainly detected during the cell-cell adhesion phase [39]. Our results demonstrated that biofilm formation of *S*. *epidermidis* is particularly inhibited by QQ-7, most likely due to interference with PIA production (Fig 7). The effects on biofilm formation of *S*. *epidermidis* were already present during early stages of biofilm formation, more precisely during the first 3 h (Fig 6). Anti-adhesive effects based on a surface charge of QQ-7 were excluded by immobilizing inactive (heat-treated) protein not effecting biofilm formation. Inhibition of *S*. *epidermidis* biofilm formation correlated with an increased synthesis of the IcaR transcriptional repressor, which in turn presumably decreased transcription of the *icaADBC* operon. Consequently, PIA production decreased and cells were not able to stick to each other to form a mature biofilm. Recently, Xue *et al*. [40] demonstrated that AI-2 increases biofilm formation of *S*. *epidermidis* RP62A by down regulating the transcription of *icaR* resulting in induced expression of the *icaADCB* operon and consequently increased PIA synthesis leading to enhanced biofilm formation. Based on our findings, we propose that QQ-7 interferes with AI-2 of *S*. *epidermidis*. Assumed quenching of the *luxS* synthesized signaling molecule AI-2 (reduction of 4-hydroxy-2,3-pentanedione-5-phosphate(P-DPD, open AI-2 form) to 3,4,4-trihydroxy-2-pentanone-5-phosphate) due to the oxidoreductase of QQ-7 might lead to increased *icaR* expression and decreased transcription of the *icaADBC* operon, ultimately negatively affecting biofilm development of *S*. *epidermidis*. Our study pointed out that biofilm formation of the opportunistic pathogens *C*. *albicans* and *S*. *epidermidis* was indeed affected by interferring their underlying QS systems. Given that the increase in drug-resistant bacteria and fungi challenges the development of novel antimicrobial drugs, it gets more and more important that in particular, drugs that efficiently target biofilm formation of opportunistic pathogens in the clinical environment, especially in biomaterial-related infections are developed [41, 42]. Here, a special challenge is combating multi-species biofilms, which are excessively associated with a high mortality rate, and often requiring the removal of the infected medical device [43]. However, removal of infected devices in less accessible locations, such as orthopedic joints or heart valves, or in patients with a reduced health condition might be impossible [44]. Besides other novel strategies for controlling biofilms in these environments such as enhancement of antimicrobial penetration with use of bioacoustics and bioelectric effects [45], modulation of biofilm-promoting genes [46] and changing adhesion properties of surfaces [47], the inhibition of microbial QS systems seems to be a promising strategy for biofilm inhibition and prevention of microbial pathogenicity and virulence [18, 48]. The present study indicates that QQ molecules might even have the potential to inhibit mixed biofilms with species from different domains of life.

## Conclusion

We identified two novel naturally occurring QQ proteins derived from a metagenomic library, which are able to inhibit *S*. *epidermidis* and *C*. *albicans* biofilm formation. It is notable that both QQ proteins significantly altered biofilm formation of two opportunistic pathogens belonging to different domains of life. Moreover, the QQ proteins most likely interfered with completely different QS signaling pathways. The fact that the immobilized proteins efficiently affected biofilm formation over several hours might indicate their great potential to combat biofilm infections by indwelling devices. In particular, QQ-7 showed significant effects on biofilm formation of both individual organisms targeting different QS systems in different domains of life, thus it can be assumed that a mixed biofilm is affected by the QQ protein. Further experiments have to elucidate their actual potential for application as novel anti-biofilm drug for combating mono- as well as muli-species biofilm infections, e.g. caused by indwelling medical devices.

## Methods

### Strains and culture media

*Candida albicans* strain DSM No. 11225 (DSMZ, Braunschweig, Germany) was grown in yeast extract peptone dextrose (YPD; 2% (w/v) tryptone, 1% (w/v) yeast extract, 2% (w/v) glucose) at 30°C. The bacterial strain *S*. *epidermidis* RP62A DSM No. 28319 (DSMZ, Braunschweig, Germany) [49] was used in this study. The strain was grown overnight in Tryptic Soy Broth (TSB) [50] medium at 37°C.

### Purification and immobilization of QQ proteins on coverslips and in multiwell plates

QQ-5 and QQ-7 have been recently identified in a metagenomic library using reporter strains AI1-QQ.1 and AI2-QQ.1 [18, 19]. Expression and purification of QQ proteins as Maltose Binding Protein (MBP)-fusions were performed as recently described in [18]. Pretreated multiwell plates (12 well MWPs) as well as coverslips were coated with ethyleneimine polymers (PEI, 25.000, fluorescein labeled) according to the previously published Layer-by-Layer method (Surflay Nanotec, Berlin/Germany) [23]. Covalent immobilization of proteins was performed as described in [18] using 83.3 μg/mL of the respective QQ protein (MBP, MBP-QQ-5, MBP-QQ-7).

### Bacterial biofilm formation assay

*S*. *epidermidis* RP62A overnight cultures were used for *in vitro* biofilm assays in 96 well microtiter plates (MTPs). 3 x 10^8^ cells/mL were washed three times in 3% (w/v) TSB. 200 μL of washed cells in TBS/well were supplemented with 10, 50 and 100 μg/well of purified proteins QQ-5 or QQ-7. Each combination was performed in triplicates. MTPs were closed with gas-permeable membranes (Breathe-Easy®, Diversified Biotech, Dedham, USA) and gently shaken at 80 rpm and 37°C. After 24 h, MTPs were used for crystal violet assay as described in [18] according to the protocol of [21] to calculate biofilm proportions.

### Fungal biofilm formation assay

*C*. *albicans* cells (10^9^ cells/mL) were grown in 12 well MWPs in presence of immobilized proteins MBP (control), MBP-QQ-5 and MBP-QQ-7 in 5 mL YPD for up to 24 h without shaking at 30°C. Three biological replicates and four independent technical replicates were performed. After 2, 4, 6, 8 and 24 h incubation, cultures were monitored by phase-contrast microscopy using Zeiss Axioskop 45 14 87 (Zeiss, Jena/Germany). Additionally, *C*. *albicans* cultures incubated in the presence of immobilized QQ proteins (83.3 μg/mL) for 0, 2, 4 and 6 h were taken to determine relative transcript levels of key gene *dpp3*.

### Biofilm formation in flow chambers

QQ active purified proteins as well as heat treated proteins (30 min at 65°C) were immobilized to coverslips. Coverslips with immobilized protein (see above) and coverslips without protein were inserted into flow chambers and sealed with polyvinylsiloxane (President Light Body, Coltène/Whaledent AG, Altstätten/Switzerland). Chambers (total volume 1.3 ml) were attached to a peristaltic pump (Ismatec ISM931C, Wertheim/Germany) and equilibrated with medium for 2 h. *S*. *epidermidis* RP62A overnight cultures were adjusted to 4 x 10^8^ cells, washed twice with 3% TSB and inoculated into the flow chambers. After 1 h without flow, biofilms were grown in 3% TSB at a flow rate of 30 mL/h at 37°C for 3 (short-term) or 20 h (long-term). Subsequently all samples were stained, visualized and microscopically analyzed.

### Confocal laser scanning and scanning electron microscopy

For confocal laser scanning microscopy (CLSM), biofilms were stained with the FilmTracerTM LIVE/DEAD® Biofilm Viability Kit (Life Technologies, Darmstadt/Germany) according to the manufacturer's protocol and visualized using a LSM 700 microscope (Zeiss, Jena/Germany). Biofilm thickness and volume were determined using IMARIS Software (Bitplane AG, Zurich/Switzerland). Subsequently, samples were prepared for Scanning Electron Microscopy (SEM). Samples were fixed in 2.5% glutaraldehyde overnight at 4°C. After fixation, samples were dehydrated in 15, 25, 40, 55, 70, 85, 95 and 100% ethanol each for 20 min [51] and critical point dried (Polaron CPD E3000 combined with Heater/chiller E4860). The dried samples were sputter-coated with 5 nm gold/palladium and images captured using SEM S-4800 (Hitachi, High-Technologies, Krefeld/Germany).

### Quantitative (q)RT-PCR

*C*. *albicans* (600 μL) incubated in MWPs in presence of immobilized MBP, QQ-5 and QQ-7 for 0, 2, 4 and 6 h (see “Fungal biofilm formation assay”) was used for extraction of mRNA using the illustra™ QuickPrep Micro mRNA Purification Kit (GE Healthcare, Freiburg/Germany). *S*. *epidermidis* (3 mL, 9 x 10^8^ cells) treated with MBP or QQ-7 was used for RNA isolation with RNeasy Mini Kit (Qiagen, Hilden/Germany) according to the manufacturer's protocol. 5 ng *C*. *albicans* cDNA synthesized with Fermentas First Strand cDNA Synthesis Kit (Fermentas, Darmstadt/Germany) were treated with DNase I with addition of RiboLock™ RNase Inhibitor (Fermentas, Darmstadt/Germany). Before use of cDNA in quantitative PCR, samples were purified using NucleoSpin Gel and PCR Clean-up kit (Macherey-Nagel, Düren/Germany). *S*. *epidermidis* RNA was precipitated using glycogen prior to DNase I treatment. qRT-PCR analyses were performed with three independent RNA preparations and each three technical replicates on the Applied Biosystems 7300 Real-Time PCR System (Thermo Fisher Scientific, Waltham, Massachusetts, USA) using the QuantiTect SYBR® Green RT-PCR Kit (Qiagen, Hilden/Germany) and primers for *dpp3* of *C*. *albicans* and *gyrB*, *icaR*, *lrgA* and *rnaIII* of *S*. *epidermidis* listed in Table 3. Fold changes in transcript abundances for *dpp3*, *icaR*, *lrgA* and *rnaIII* were determined by comparison with the threshold cycle (Ct) of transcripts of the control gene *actin* for *dpp3* and *gyrB* for *icaR*, *lrgA* and *rnaIII*. The fold change in the abundance of a transcript was calculated using the equation fold change = 2 –^ΔΔCt^ as described [52, 53].

**Table 3:** Oligonucleotide primers used for qRT-PCR analyses.

Target gene	Oligonucleotide primers sequence (5’ to 3’)		Reference
	forward	reverse	
***dpp3***	CTCCTTCGGGTCATTCATCAA	GATTACCACCAAACCAAGGGA	This study
***actin***	GATTTTGTCTGAACGTGGTAACAG	GGAGTTGAAAGTGGTTTGGTCAATAC	[54]
***icaR***	GTGTTGAGAATTGTTTCAATTACTTTAT	CTCTCATCAACGTCGAATATAAAT	This study
***lrgA***	CAAGCATTAACGATTGCAGTGATTTTAC	CAACTTGACCTAATTTCACAATGCC	This study
***rnaIII***	CAATCGGTGATTTAGTAAAATGGA	GTTGGGATGGCTCAACAAC	[55]
***gyrB***	ATCAACATCGGCATCAGTCA	GCATTTGGTACGGGTATTGG	[56]

### Statistical analysis

Statistics were performed with GraphPad Prism 6 software (GraphPad, San Diego/USA) with differences *P < 0.05, **P < 0.01 and ***P < 0.001 considered significant.
